# Association Between *FoxO1*, *A2M*, and *TGF-β1*, Environmental Factors, and Major Depressive Disorder

**DOI:** 10.3389/fpsyt.2020.00675

**Published:** 2020-07-10

**Authors:** Mingzhe Zhao, Lu Chen, Zhengxue Qiao, Jiawei Zhou, Tianyu Zhang, Wenxin Zhang, Siyuan Ke, Xiaoyun Zhao, Xiaohui Qiu, Xuejia Song, Erying Zhao, Hui Pan, Yanjie Yang, Xiuxian Yang

**Affiliations:** ^1^Psychology Department, Public Health Institute, Harbin Medical University, Harbin, China; ^2^Department of Endocrinology, Peking Union Medical College Hospital, Beijing, China

**Keywords:** *FoxO1*, *A2M*, *TGF-β1*, depression, G×E interaction, polymorphism

## Abstract

**Introduction:**

Investigations of gene-environment (G×E) interactions in major depressive disorder (MDD) have been limited to hypothesis testing of candidate genes while poly-gene-environmental causation has not been adequately address. To this end, the present study analyzed the association between three candidate genes, two environmental factors, and MDD using a hypothesis-free testing approach.

**Methods:**

A logistic regression model was used to analyze interaction effects; a hierarchical regression model was used to evaluate the effects of different genotypes and the dose-response effects of the environment; genetic risk score (GRS) was used to estimate the cumulative contribution of genetic factors to MDD; and protein-protein interaction (PPI) analyses were carried out to evaluate the relationship between candidate genes and top MDD susceptibility genes.

**Results:**

Allelic association analyses revealed significant effects of the interaction between the candidate genes *Forkhead box* (*Fox*)*O1, α2-macroglobulin* (*A2M*), and *transforming growth factor* (*TGF*)*-β1* genes and the environment on MDD. Gene-gene (G×G) and gene-gene-environment (G×G×E) interactions in MDD were also included in the model. Hierarchical regression analysis showed that the effect of environmental factors on MDD was greater in homozygous than in heterozygous mutant genotypes of the *FoxO1* and *TGF-β1* genes; a dose-response effect between environment and MDD on genotypes was also included in this model. Haplotype analyses revealed significant global and individual effects of haplotypes on MDD in the whole sample as well as in subgroups. There was a significant association between GRS and MDD (P = 0.029) and a GRS and environment interaction effect on MDD (P = 0.009). Candidate and top susceptibility genes were connected in PPI networks.

**Conclusions:**

*FoxO1*, *A2M*, and *TGF-β1* interact with environmental factors and with each other in MDD. Multi-factorial G×E interactions may be responsible for a higher explained variance and may be associated with causal factors and mechanisms that could inform new diagnosis and therapeutic strategies, which can contribute to the personalized medicine of MDD.

## Introduction

Major depressive disorder (MDD) is the most common psychiatric disorder and is associated with high morbidity, mortality, costs, and risk of suicide (1–3). The heritability of MDD is approximately 30%–40% (4) and may be greater for recurrent, early-onset disease (5). Establishing the contribution of genetics to MDD can lead to more accurate and timely diagnosis and clinical management, which has significant public health implications.

Studies on main genetic effects in MDD have reported conflicting findings (6). One reason for this is that risk of MDD is polygenic, with many susceptibility genes exerting small effects (7). Additionally, genotypic variations among individuals may increase the risk of MDD only upon exposure to adverse environmental factors, a phenomenon known as gene-environment (G×E) interaction (8). The association between candidate susceptibility genes and disease occurrence has mainly been predicated on the observation that genetic variants not only increase MDD risk but can also explain the underlying biological and molecular mechanisms (9). However, such hypothesis-driven approaches to the study of MDD etiology can only detect a fraction of genetic variants (8). On the other hand, a hypothesis-free approach requires enormous datasets that include both exposure [e.g., stressful life events (10, 11) and childhood adversities (9)] and phenotype (MDD) data. To this end, only three genome-wide environmental interaction studies has been carried out (12–14). In a recent report, a novel omics-based approach was used to identify genetic variants for genome-wide G×E interaction studies based on cross-species and -tissue biological prioritization strategies. However, genomics approaches usually cannot identify causal variants or genes (15). Integrating multiple omics approaches can provide a more comprehensive view of the biology of MDD.

Three candidate MDD susceptibility genes [*Forkhead box* (Fox)*O1*, *transforming growth factor* (TGF)*-β1*, and *α2 macroglobulin* (A2M)] that are ideal candidates for G×E interaction analyses were previously identified using an omics-based approach. It has been suggested that environmental and genetic factors contribute equally to the development of mental illness (16). Distal environmental risk factors such as family history (FH) and culture are important because they increase vulnerability to proximal factors; (17) however, the latter are more relevant for G×E investigations since they are more likely to meet the criteria for risk factors and lend themselves to biologically plausible hypotheses regarding their effects on specific neural systems that underlie psychopathological symptoms.

Genome-wide association studies (GWAS) have identified more than 100 genetic variants associated with MDD (18). Each of these has a small effect and the number of associated genetic markers increases with sample size. However, the utility of individual genetic markers for MDD risk prediction is uncertain (19). A multilocus genetic risk score (GRS)-based analysis has been proposed for combining the relatively small effects of single genes to better assess the complex relationship between genetic markers and MDD (20). GRS analyses have shown that including more weakly associated genetic variants improves the prediction of mental illness risk (21).

Proteins interact to mediate many cellular processes; disrupting one subunit of a protein complex can lead to direct and indirect functional consequences in various diseases and physiological conditions (22). For a specific illness, interactions between proteins encoded by susceptibility genes tend to be more frequent than those between random proteins, as revealed by protein-protein interaction (PPI) network analyses (22).

In the present study, we investigated the association between *FoxO1*, *TGF-β1*, and *A2M* genes and MDD in a large population. G×E, G×G, G×G×E, and environment × environment (E×E) interactions were analyzed in the context of MDD. We also assessed haplotypes of these three genes and the relationship between haplotype and environment. We used a GRS approach to calculate the combined effects of these three genes on the prediction of MDD and the GRS–environment interaction (GRS×E) in MDD. Finally, we examined FoxO1, TGF-β1, and A2M PPI networks to clarify their interactions with each other and with other MDD-related proteins.

## Materials and Methods

### Study Population

From November 2014 to December 2018, 800 MDD patients (564 women and 236 men) were recruited for the study (mean age: 45.64 ± 14.10 years) along with 800 age- and sex-matched control subjects from the same geographic area in Northern China. All subjects were of Chinese Han ethnicity and provided written, informed consent before participating in the study. The study protocol was approved by the ethics committee of Harbin Medical University.

### Independent Measures

Participants completed three questionnaires: a socio-demographic questionnaire, the Chinese version of the 24-item Hamilton Rating Scale for Depression (HRSD-24), and the Life Events Scale (LES). The self-rating socio-demographic questionnaire was adapted from the version developed by the Epidemiology Department of Harbin Medical University and was used to collect detailed background information on family psychiatric history, childhood trauma history, and socioeconomic background. The HRSD-24 (23) is widely used to measure depression symptoms; (24–26) a number of patients above the threshold (21 points) were included in the study. The LES questionnaire for measuring negative life events contained 48 items in three dimensions: family life (28 items), work-related problems (13 items), and social and other aspects (seven items). The LES was scored based on the occurrence/absence (1 and 1, respectively) and frequency (0 or more) of SLEs (26, 27).

### Genotyping

Single nucleotide polymorphisms (SNPs) in the *FoxO1* (rs2297626, rs7319021, rs28553411, rs17592468, and rs17592371), *A2M* (rs10492115, rs226415, rs10842849, rs11048839, rs10842847, and rs669), and *TGF-β1* (rs2317130, rs1800469, rs12983775, rs12462166, and rs2241715) genes were selected for genotyping, the candidate SNPs comprised most of the allelic variants with r^2^ > 0.8 in the Asian population. Genomic DNA was extracted from venous blood samples using the AxyPrep Blood Genomic DNA Miniprep kit (Axygen, Union City, CA, USA). Polymerase chain reaction (PCR) was performed in a reaction volume of 2 μl DNA, 7.5 μl 2× PCR mix, 2 μl primer mix, 0.2 μl *Exo*I enzyme (Fermentas, Burlington, ON, Canada), 0.7 μl *Exo*I buffer (Fermentas), and 0.8 μl FastAP enzyme (Fermentas). Amplification conditions were as follows: at 95°C for 3 min; 35 cycles of 94°C for 15 s, 55°C for 15 s, and 72°C for 30 s; and 72°C for 3 min. PCR products were purified by incubation with *Exo*I and FastAP at 37°C for 15 min and at 80°C for 15 min. Extension conditions were as follows: 96°C for 1 min, and 30 cycles of 96°C for 10 s, 52°C for 5 s, and 60°C for 30 s. Extension products after denaturation at 95°C for 3 min were analyzed by DNA sequencing (Applied Biosystems, Foster City, CA, USA).

### Statistical Analysis

Haploview v.4.0 software was used to assess Hardy-Weinberg Equilibrium (HWE) and pairwise linkage disequilibrium (LD) and calculate minimal allele frequency (MAF) for genotyped polymorphisms (28). The χ^2^ test/Fisher’s exact test was used to analyze differences in the distributions of independent variables. The Bonferroni method was adopted for multiple-testing correction. Associations between phenotype and independent variables were analyzed by multivariable logistic regression, with polymorphisms scored as 0, 1, or 2 depending on the carrier status of the minor allele, sex, family history (FH), occurrence/absence or number of SLEs and CAs, and interaction effects (E×E/G×E/G×G/G×G×E/GRS×E). Allele-carrier status and dose-response effect of environmental factors were also determined. Hierarchical regression analysis was performed using RStudio v.1.1.423 software. Alpha levels were corrected with the number of polymorphisms of candidate genes (five polymorphisms for *FoxO1* and TGF*-β1*; six polymorphisms for *A2M*), so that the p values correction was 0.05/5 = 0.01 for *FoxO1* and TGF*-β1*, 0.05/6 = 0.0083 for A2M. Study power was calculated with QUANTO 1.2.4 (http://hydra.usc.edu/gxe/).

Haplotype analysis was performed using UNPHASED v.3.0.11 software (29). Maximum likelihood haplotype frequencies in the study population were assessed with the expectation maximization algorithm. Rare haplotypes with a frequency < 1% were excluded from analyses. The global association and effect of individual haplotypes on phenotype were analyzed in the total sample and in four subgroups classified according to positive/negative environmental factors (SLEs/CAs). The significance level of the global association was determined with the likelihood ratio test. Individual effects of each haplotype, i.e., the difference in effect between a haplotype and all others pooled together, were computed with the score test. A permutation analysis was conducted to assess the reliability of the results; 1,000 random permutations were set to generate empirical P values. Permuted P < 0.05 was considered significant in the haplotype analyses.

### GRS Analysis

To estimate the cumulative contribution of genetic factors to MDD in an individual by taking into account the reported risk alleles, we used the predictABEL package in RStudio software to compute weighted GRS with the following formula:

$$\text{GRS}=\Sigma_{i=1}^{k}\beta_{i}N_{i}$$

where GRS is the sum of the effect estimates, k is the number of independent genetic variants with strong association as risk predictors, β_i_ is the weighted coefficient from logistic regression analysis, and N_i_ is the number of risk alleles for each locus. The association between GRS and MDD was evaluated by logistic regression analysis of FH, sex, GRS, SLEs/CAs, and the interaction between GRS and SLEs/CAs.

### Population Stratification Analysis

Population stratification analysis was performed to eliminate the possibility of false-positive associations using STRUCTURE v.2.3.4 software (http://pritch.bsd.uchicago.edu/structure.html). *FoxO1*, *A2M*, and *TGF-β1* genotype data were used for population stratification analysis of the third-stage sample set. Sixteen SNPs in these genotype datasets were obtained from 311 samples derived from the 1000 Genomes Project, including 99 samples from the Yoruba population of Ibadan, Nigeria (YRI), 109 samples from Chinese Han in Beijing (CHB), and 103 samples from northern and western Europe and the United States (CEU) (1000 Genomes Project Phase 3 at www.internationalgenome.org/data-portal/sample). STRUCTURE assumes that there are K populations in the dataset. The admixture and correlated frequency models had a burn-in length of 10,000 and 10,000 Markov chain Monte Carlo repeats and took into consideration immigration and geography-based genetic isolation. The program was run several times at each K value from 2 to 6 to obtain consistent results.

### PPI Analysis

We investigated whether *FoxO1*, *A2M*, and *TGF-β1* genes interact with each other and are involved in the PPI network containing protein products of the top MDD susceptibility genes identified by a recent GWAS (30–32). The PPI network comprises nodes and edges representing protein and physical interactions, respectively. Proteins encoded by MDD susceptibility genes were used as seed proteins. STRING v.11.0 software (https://string-db.org/cgi/input.pl) (33) was used to reconstruct the PPI network.

## Results

### Descriptive Statistics

**Figure 1:**
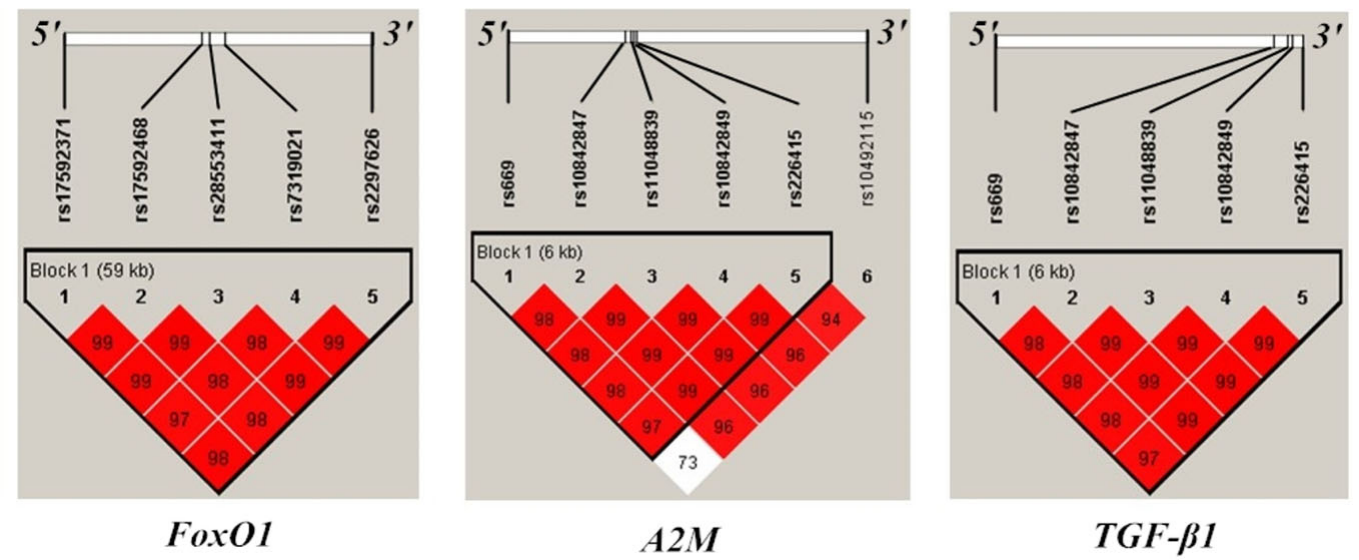
Position and linkage disequilibrium (LD) map of genotyped polymorphisms in FoxO1, A2M, and TGF-β1gene. Pairwise LD statistics in examined genes were calculated with Haploview. Squares are bright red if the |D’| value is high, that is, LD is strong.

Demographic information on the study population is shown in **Table 1**. Genetic markers were successfully genotyped at a rate > 95%. No significant deviation from HWE was observed, and MAF was > 5% for each polymorphism (**Supplemental Table S1**). Pairwise LD D’ values are shown in **Figure 1**.

**Table 1:** Summarized frequencies of demographic and independent variables.

Variables	Frequencies
Gender	
Female	1,023 (63.9%)
Male	578 (36.1%)
Mean age	44.28 (s.d. 11.97)
Exposure to CAs	
No	1,412 (88%)
Yes	189 (12%)
Exposure to SLEs	
No	820 (51%)
Yes	781 (49%)
1	554 (70%)
2	74 (9%)
3 or more	153 (21%)
Family history of psychological problems among first-degree relatives	
FH−	1,440 (90%)
FH+	161 (10%)

FH, family history.

### Association Between Independent Variables and MDD

Two polymorphisms of *FoxO1* (rs17592371 and rs2297626), two of *A2M* (rs669 and rs226415), and one of *TGF-β1* (rs12462166) were significantly associated with MDD (**Table 2**). Family history of MDD (χ^2^ = 54.823, P < 0.0001), occurrence (χ^2^ = 406.993, P <0.0001), and number (χ^2 =^ 781.522, P < 0.0001) of SLEs were related to MDD. The results remained robust after Bonferroni correction (**Table 2**). The power for association study of *FoxO1* (rs17592371 and rs2297626) was 87.43% and 90.45% and the power of *TGF-β1* (rs12462166) was 86.55%.

**Table 2:** Association of Study Variables with MDD.

Variable	Subgroup	Number		χ^2^ Value	P Value	P-Bonferroni
**FoxO1**		Case	Control			
rs17592371	CC	293	359	9.790	0.007	0.035
	CT	387	318			
	TT	120	123			
rs2297626	AA	293	356	11.424	0.003	0.015
	AG	382	321			
	GG	125	123			
**A2M**						
rs669	AA	694	644	Fisher’s	0.001	0.006
	AG	101	152			
	GG	5	4			
rs226415	AA	6	15	10.395	0.005	0.03
	AG	168	158			
	GG	626	627			
**TGF-β1**						
rs12462166	CC	132	187	13.679	0.001	0.005
	CT	454	438			
	TT	214	175			
**FH**						
	Yes	125	36	54.823	<0.001	<0.001
	No	675	765			
**SLEs presence**						
	Yes	592	189	406.993	<0.001	<0.001
	No	208	612			
**SLEs amount**						
	0	15	613	781.522	<0.001	<0.001
	1	438	116			
	2	38	36			
	3 or more	110	36			

FH, family history; SLEs, stressful life events.

### Interaction Between SLEs and Candidate Gene Genotypes in MDD

**Figure 2:**
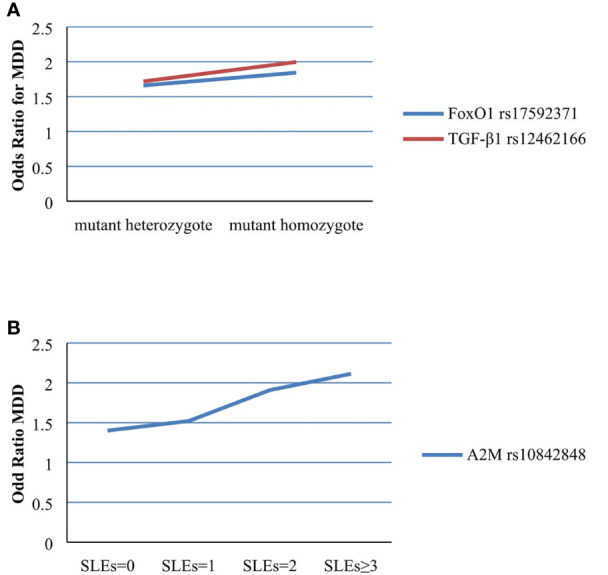
**(A)** Effect of SLEs on MDD in different genotypes of rs17592371 in FoxO1 and rs12462166 in TGF-β1. **(B)** Effect of rs10842848 in A2M on MDD in different number of SLEs.

The two *FoxO1* (rs17592371 and rs28553411), three *A2M* (rs10842847, rs10842849, and rs226415), and two *TGF-β1* (rs12462166 and rs12983775) gene polymorphisms showed significant interactions with environmental factors (**Table 3**). In the hierarchical regression analysis, the effect of SLEs on MDD was stronger for the TT genotype (β = 0.612, SE = 0.25, z = 2.450, P = 0.014) than for the TC genotype (β = 0.508, SE = 0.176, z = 2.878, P = 0.003) of *FoxO1* rs17592371; and for the GG genotype (β = 0.692, SE = 0.291, z = 2.379, P = 0.017) as compared to the GT genotype (β = 0.542, SE = 0.166, z = 3.258, P = 0.001) of *TGF-β1* rs12462166 (**Figure 2A**). A dose-response effect of the interaction between number of SLEs and genotypes in MDD was only observed for *A2M* rs10842848, which did not interact with an SLE of 0 (β = 0.099, SE = 0.170, z = 0.586, P = 0.558) but showed interactions with an SLE of 1 (β = 0.420, SE = 0.179, z = 2.339, P = 0.019), 2 (β = 0.647, SE = 0.278, z = 2.330, P = 0.019), and 3 or more (β = 0.749, SE = 0.295, z = 2.535, P = 0.011). That is, high exposure to SLEs was related to an increase in MDD risk (**Figure 2B**).

**Table 3:** Effect of Candidate Genes, Environment, and Their Interaction on MDD.

Dependent Variables	β	SE	z	p Value
**FoxO1**				
rs17592371	−0.051	0.079	−0.651	0.515
CAs	0.521	0.235	2.215	0.026
rs17592371×CAs	0.589	0.250	2.352	0.018
rs17592371	−0.156	0.121	−1.285	0.198
SLEs	1.686	0.170	9.905	<0.001
rs17592371×SLEs	0.468	0.171	2.739	0.006
rs28553411	−1.210	0.148	−8.136	<0.001
CAs	1.622	0.223	7.247	<0.001
rs28553411×CAs	0.616	0.202	3.047	0.002
rs17592371	1.881	0.393	4.781	<0.001
rs28553411	−1.746	0.421	−4.141	<0.001
rs17592371×rs28553411	0.275	0.108	2.547	0.010
rs17592371	1.133	0.679	1.668	0.095
rs28553411	−1.219	0.679	−1.796	0.072
CAs	−0.083	0.202	−0.413	0.679
rs17592371×rs28553411×CAs	0.321	0.109	2.922	0.003
rs17592371	1.104	0.721	1.531	0.125
rs28553411	−1.226	0.721	−1.700	0.089
SLEs	1.704	0.143	11.842	<0.001
rs17592371×rs28553411×SLEs	0.211	0.087	2.409	0.016
**A2M**				
rs10842847	−0.142	0.120	−1.181	0.238
CAs	−0.133	0.183	−0.725	0.468
rs10842847×CAs	0.894	0.371	2.409	0.016
rs10842847	−0.206	0.176	−1.169	0.242
SLEs	1.152	0.124	9.273	<0.001
rs10842847×SLEs	0.711	0.253	2.811	0.004
rs10842849	−0.166	0.121	−1.372	0.170
CAs	−0.129	0.184	−0.704	0.481
rs10842849×CAs	0.988	0.374	2.639	0.008
rs10842849	−0.172	0.176	−0.978	0.327
SLEs	1.283	0.124	10.309	<0.001
rs10842849×SLEs	0.608	0.254	2.397	0.016
rs226415	2.310	0.194	1.201	0.023
SLEs	2.310	0.134	17.136	<0.001
rs226415×SLEs	−0.697	0.251	−2.775	0.005
rs10842849	0.052	0.615	0.085	0.932
rs226415	0.700	0.673	1.039	0.298
rs10842849×rs226415	−0.676	0.289	−2.341	0.019
rs10842849	−0.123	0.625	−0.197	0.843
rs226415	−0.060	0.631	−0.095	0.924
CAs	−0.141	0.183	−0.768	0.442
rs10842849×rs226415×CAs	1.132	0.378	2.989	0.002
**TGF-β1**				
rs12461266	−0.206	0.082	−2.493	0.012
CAs	−0.159	0.277	−0.574	0.565
rs12461266×CAs	0.629	0.202	3.106	0.001
rs12461266	−0.276	0.124	−2.218	0.026
SLEs	1.488	0.193	7.667	<0.001
rs12461266×SLEs	0.527	0.167	3.146	0.001
rs12983775	0.066	0.126	0.520	0.603
SLEs	2.363	0.204	11.563	<0.001
rs12983775×SLEs	−0.479	0.167	−2.857	0.004

CAs, childhood adversities; SLEs, stressful life events.

We evaluated the G×G interaction between *FoxO1* rs17592371 and rs28553411, between the *TGF-β1* rs12462166 and rs12983775, and among *A2M* rs10842847, rs10842849, and rs226415 as well as the three-way interaction between the two genetic markers that were significant in the G×G interaction analysis and LES (**Table 3**). The interaction between the two polymorphisms of the *FoxO1* gene was significant (β = 0.275, SE = 0.108, z = 2.547, P = 0.010), as was the interaction between those of the *A2M* gene (β = −0.676, SE = −0.289, z = −2.341, P = 0.019). The effects of three-way interaction (G×G×E) on MDD were also significant for *FoxO1* (G×G×SLE: β = 0.211, SE = 0.087, z = 2.409, P = 0.016; G×G×CA: β = 0.321, SE = 0.109, z = 2.922, P = 0.003) and *A2M* (G×G×CA: β = 1.132, SE = 0.378, z = 2.989, P = 0.002) (**Table 3**). There results remained significant after controlling for family history and sex in the regression model. The effect of E×E interaction on MDD was significant (β = 0.746, SE = 0.312, z = 2.392, P = 0.016).

### Haplotype Analysis

Haplotype analysis was performed for the total sample and subgroups. In the former, 12 haplotypes of five *FoxO1* gene polymorphisms, 15 haplotypes of five *TGF-β1* gene polymorphisms, and 14 haplotypes of *A2M* gene polymorphisms had a frequency > 1%. The haplotypes of the *FoxO1* gene showed a significant global association (χ^2^ = 19.732, df = 13, P_global_ < 0.001) and the less common haplotype (G-A-G-C-T-A) of the *A2M* gene was significantly associated with MDD (χ^2^ = 4.931, P_effect_ = 0.026). In subgroup samples, haplotypes of the *A2M* gene within the SLE occurrence group (χ^2^ = 21.267, df = 11, P_global_ = 0.030) and haplotypes of *FoxO1* (χ^2^ = 41.792, df = 11, P_global_ < 0.001) and *TGF-β1* (χ^2^ = 34.531, df = 14, P_global_ = 0.001) genes within the CA occurrence group were significant in the global association. Two less common haplotypes (G-A-A-C-G-G and G-A-G-C-T-A) of the *A2M* gene within the SLE occurrence group (χ^2^ = 5.815, P_effect_ = 0.015; χ^2^ = 5.814, P_effect_ = 0.015) and a less common *A2M* haplotype (G-A-G-C-T-A) in the CA occurrence group (χ^2^ = 3.947, P_effect_ = 0.046) were associated with MDD.

### GRS Analysis

After controlling for FH and sex, logistic regression revealed that the 16 SNP GRSs comprising risk variants were associated with MDD (β = 0.022, SE = 0.010, z = 2.180, P = 0.029). Interaction analyses indicated that GRSs interacted with SLEs in MDD (β = 0.054, SE = 0.021, z = 2.577, P = 0.009).

### Population Stratification Analysis

The combined population of YRI, CHB, and CEU exhibited obvious stratification (**Supplemental Figure S1A**). In the triangle chart with K = 3, each angle represented a potentially independent ancestry and the different colored dots represented individuals in assumed population components that did not cluster with the same color in the triangle, indicating that there was no significant stratification in our sample (**Supplemental Figure S1B**). Consistent results were obtained with K values ranging from two to six. Thus, population stratification was unlikely to be a confounding factor in this study.

### PPI Analysis

**Figure 3:**
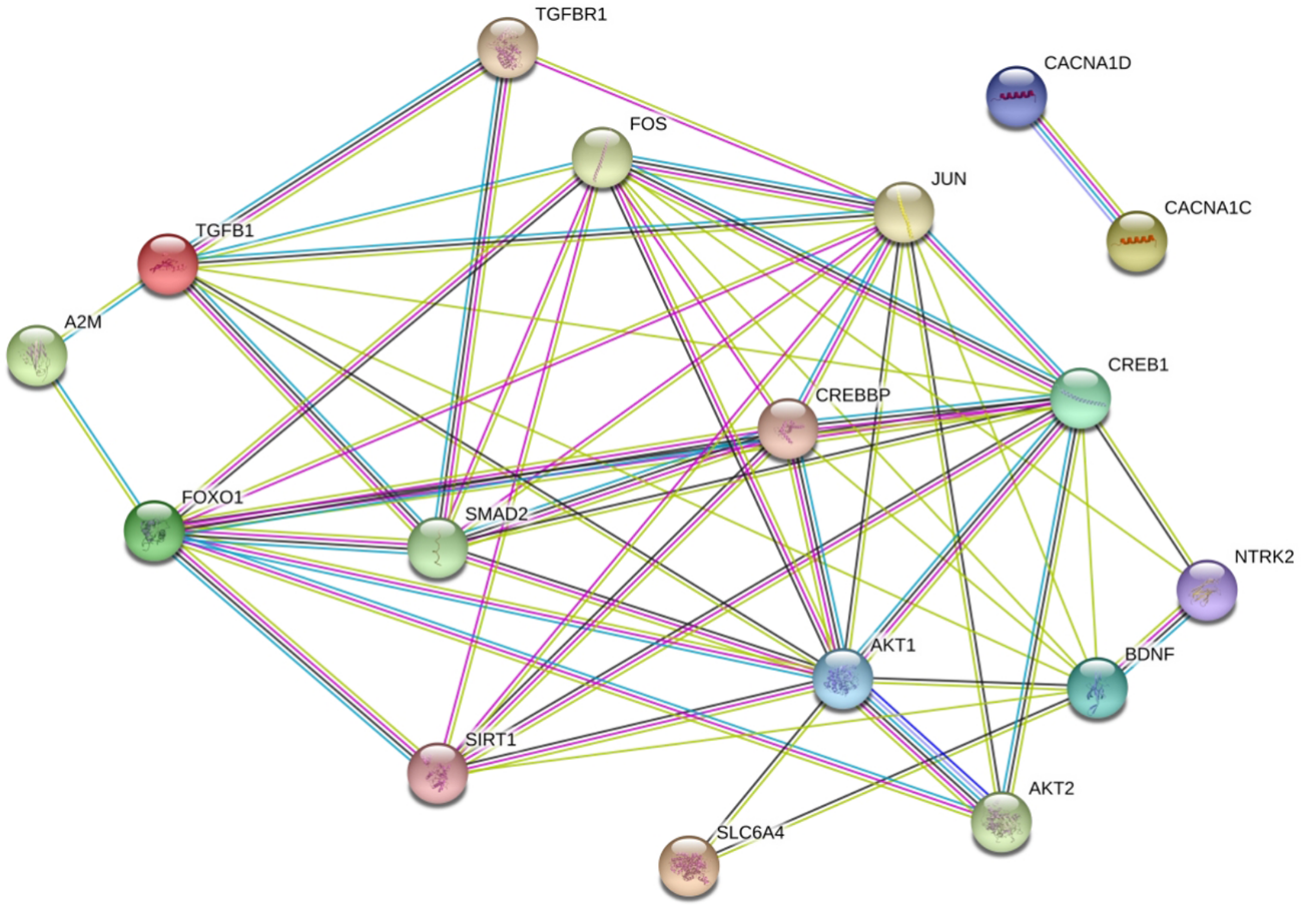
Proteins encoded by FoxO1, A2M, and TGF-β1 in a densely interconnected protein-protein interaction (PPI) network formed by susceptibility genes of MDD. (33) The amaranthine lines indicate known PPIs that have been experimentally determined, and the light blue lines indicate known PPIs from curated databases. The green lines indicate predicted interactions from gene neighborhood, the dark blue lines indicate predicted interactions through gene co-occurrence and the black lines indicate known PPIs from co-expression. The light green lines indicate predicted PPIs through text mining.

We detected interactions among FoxO1, A2M, and TGF-β1 proteins and the protein products of multiple MDD risk genes. The top susceptibility genes except for the *calcium voltage-gated channel subunit α1 C* gene, identified in GWASs of MDD (30–32) formed a densely interconnected PPI network (**Figure 3**) that included FoxO1, A2M, and TGF-β1. Interestingly, A2M was found to directly interact with FoxO1 and TGF-β1. TGF-β1 directly interacted with FOS, JUN, cyclic AMP response element-binding protein (CREB) 1, brain-derived neurotrophic factor, AKT1, and Mothers against decapentaplegic homolog (SMAD) 2; and FoxO1 directly interacted with FOS, JUN, CREB binding protein, SMAD2, AKT1, AKT2, and sirtuin 1. These factors have been implicated in MDD genetic risk studies (34–38).

## Discussion

The interaction between genetic markers and environment determines the risk of MDD (8). However, most hypothesis-driven studies of MDD to date have not produced reproducible findings. In this study, we investigated candidate MDD risk genes using a hypothesis-free, omics-based, cross-tissue and -species approach. We found that *FoxO1* rs17592371 and rs2297626, *A2M* rs669 and rs226415, and *TGF-β1* rs12462166 alleles were associated with MDD. We also found that *FoxO1* rs17592371 and rs28553411, *A2M* rs10842847, rs10842849, and rs226415, and *TGF-β1* rs12462166 and rs12983775 interacted with the environment in MDD, suggesting a potential relationship between these genes and the etiology of MDD, which could be a bona fide association or the result of linkage. A known allelic association between a gene and a disorder may reflect a G×E interaction, but the absence of such an association does not disqualify a gene as a candidate for disease risk. Some evidences showed that *FoxO1* could express in hippocampus and corpus striatum, *FoxO1* may contribute to the pathological process of MDD or other psychiatric disorders (18, 39). It was suggested that immune system associated with MDD and *A2M* played an important role in the immune system, which indicated that *A2M* may associate with MDD. Some studies found that the expression of *A2M* was higher in MDD patients comparing healthy people (21, 40). *TGF-β1* can restrain autoimmune response and maintain immune system stability, some studies found that *TGF-β1* could moderate the imbalance of proinflammatory cytokines and anti-inflammatory cytokines in MDD patients (15, 16). All of these evidences above were consistent with our findings.

The carrier status of the minor allele and number of SLEs were separately used to test different genotypic effects on MDD and the dose-response relationship between SLEs and MDD. The effect of SLEs on MDD was more highly significant for the TT than for the TC genotype of *FoxO1* rs17592371 and for the GG as compared to the GT genotype of *TGF-β1* rs12462166. These results indicated that mutant homozygous genotypes may be more significant than the heterozygous genotype for the effect of SLEs on MDD, as previously suggested (41, 42). We also found that exposure to SLEs was related to an increased risk for MDD, which is consistent with previous reports (42, 43), indicating that although the effect of a single environmental factor may be quite small, the cumulative effect of multiple factors may be large and that strong effects typically result from a chain of related events rather than a single factor.

Because gene variants can interact, we examined the SNPs that showed significant G×E interactions to investigate G×G interactions. We found that two polymorphisms in the *FoxO1* gene and the *A2M* gene showed significant interactions and that the G×G×E interaction was also significant. This was similar to a previous finding that *serotonin transporter-linked polymorphic region* (*5-HTTLPR*) gene and SNP rs140700 both interacted with the environment and with each other in MDD, and that the 5-HTTLPR×rs140700×E interaction was significant (41). Much of the genetic variation associated with a complex trait can be explained by the joint contribution of multiple genetic markers as well as environmental influence (44). G×G and G×G×E interaction analyses are considered as biologically relevant and explain the role of heritability (45–48). We also found that the E×E (CA × SLE) interaction in MDD was significant. CA increases the likelihood of SLE occurrence (49) that is, early negative experiences increase vulnerability to such events later in life, resulting in a sequential E×E interaction. For instance, mistreatment in childhood caused sensitization to the effects of specific types of SLE in adulthood (49). The present study is the first report of an E×E interaction in MDD.

Although direct associations between haplotype and psychiatric disorders have been reported (50, 51), there have been no studies demonstrating an interaction between haplotype and environmental factors. In our study, the global effect of haplotype on MDD was significant for the *FoxO1* and *TGF-β1* genes in the total sample and in subjects that had experienced CA. A significant individual effect and global effect of haplotype on MDD was found for the *A2M* gene in both the total and subgroup samples. The global effect of haplotype on MDD was always significant in combination with environmental factors. Interactions existed not only between the environment and genes, but also between the environment and haplotype in MDD.

GRS analyses have shown that the prediction of mental illness is improved by including more weakly associated genetic variants, suggesting that these influence the risk of mental disorder (21, 40). Our results showed that the association between 16 SNP GRS and MDD was significant, with a significant interaction between GRS and SLEs. Some of the 16 SNPs did not show a significant conditional or interaction effect. GRSs may detect the effects of weaker associated genetic variants (52). The results of the PPI analysis showed that FoxO1, A2M, and TGF-β1 were connected, suggesting that a wide variety of cellular processes are involved in MDD. This could explain the reason for the additive effects of *FoxO1*, *A2M*, and *TGF-β1* gene variants indicated by the GRSs. In addition, several top MDD susceptibility genes were included in this PPI network, although the clinical significance of these associations requires further investigation. The disturbances of certain cellular processes or pathways contributing to the risk of MDD has been emerging and gained evidences supporting (22, 53, 54). Constructing highly interconnected PPI networks between *FoxO1*, *A2M*, and *TGF-β1* protein and proteins of multiple defined risk genes for MDD may reveal the underlying biological mechanisms. *FoxO1*, *A2M*, and *TGF-β1* protein could participate in this PPI network, indicating their potential involvement in the common molecular network modulating the pathogenesis of MDD. It is noteworthy that *A2M* protein directly interacted with *TGF-β1* protein, indicating their connection in the biological process relevant to MDD. A recent study found that *A2M* as an inflammatory fluid proteinase scavenger could bind to a plethora of cytokines, including *TGF-β1*. (55)

We investigated the potential influence of population stratification in our samples using STRUCTURE software v.2.3.4 and the third-stage sample set, but did not find any evidence of stratification, which confirmed that the results were not affected by this confounding factor.

Our study had three major limitations. First, we did not use standard questionnaires that have confirmed reliability and validity to measure CA. Second, we did not analyze additional SNPs in our samples and the third-stage sample set, which can improve the reliability of population stratification analysis. Lastly, including a gene expression microarray analysis in our study would have allowed us to compare the transcriptomes of subjects exposed to SLEs/CAs and non-exposed individuals, which could provide more information for predicting MDD risk.

In conclusion, our data indicated that the hypothesis-free approach is useful for identifying novel genes contributing to the G×E interaction in MDD. *FoxO1*, *A2M*, and *TGF-β1* genes not only interact with environmental factors but also associate with multiple other genes in the MDD G×E interaction. *FoxO1*, *A2M*, and *TGF-β1* genes may serve the clinical diagnose and treatment of MDD by providing biomarkers, which can contribute to personalized medicine. Future studies will need to focus on the complexity of poly-gene–environmental causation to obtain more detailed insight into the etiology of MDD.

## Data Availability Statement

All datasets for this study are included in the article/**Supplementary Material**.

## Ethics Statement

The studies involving human participants were reviewed and approved by ethics committee of Harbin Medical University. The patients/participants provided their written informed consent to participate in this study.

## Author Contributions

MZ and LC conducted the statistical analyses and wrote the first draft of the manuscript. ZQ and JZ provided expertise in MDD search. TZ, WZ, and SK did the experiment. XZ, EZ, and XS collected the data. XQ and HP revised the manuscript. YY and XY designed this study and provided expertise. All authors were involved in modifying the secondary-analysis design and editing the manuscript. All authors contributed to the article and approved the submitted version.

## Funding

This study was supported by the National Natural Science Foundation of China (81773536) to YY, the Fundamental Research Funds for State Universities of Heilongjiang Province (2017JCZX23) to EZ, and China Postdoctoral Science Foundation Grant (2019M651311) to JZ.

## Conflict of Interest

The authors declare that the research was conducted in the absence of any commercial or financial relationships that could be construed as a potential conflict of interest.

The reviewer JM declared a past co-authorship with several of the authors YY, ZQ, EZ, XS, XY to the handling Editor.
