# Supplementary material for: Association Between FoxO1, A2M, and TGF-β1, Environmental Factors, and Major Depressive Disorder
Source: Front Psychiatry. 2020 Jul 10;11:675. doi: 10.3389/fpsyt.2020.00675 (PMC7394695; doi:10.3389/fpsyt.2020.00675)
Supplement: Supplementary file 1 [file Table_1.docx]

**Table S1.** Descriptive Statistic of Genotyped Polymorphisms in the FoxO1, TGF-β1, and A2M Gene

| Markers | Alleles | Region | Primer Sets | HWE | MAF |
| --- | --- | --- | --- | --- | --- |
| **FoxO1** |  |  |  |  |  |

| rs17592371 | C/T | intron | f:5- GAAATGATGAAATGGTTCCAGCC-3 | 0.834 | 0.388 |
| --- | --- | --- | --- | --- | --- |
|  |  |  | r:5- CACAGTAGAGCATGGAGTTGAAG-3 |  |  |
|  |  |  | e:5-CTGACTGACTGACTGACTGACTGACTGACTG |  |  |
|  |  |  | ACTGACTGGGGAAGAGAAAGAGGAAAA-3 |  |  |
| rs2297626 | A/G | intron | f:5-TGTAGTCTTCTAAGGAACATAGGA-3 | 0.872 | 0.392 |
|  |  |  | r:5-AAAGGCTGAACCATATAAAATAGC-3 |  |  |
|  |  |  | e:5-CTGACATGAAAAACACATTTTTGT-3 |  |  |
| rs17592468 | A/G | intron | f:5-GCTAGAGAACAAACATTCCTCCAT-3 | 0.306 | 0.394 |
|  |  |  | r:5-CATGTAGGGCAGTTGCCAATA-3 |  |  |
|  |  |  | e:5-CTGACTGAAATAAAGTCACCCTTGGGTA-3 |  |  |
| rs28553411 | A/T | intron | f:5-GGAGTAAACTTTTCAGGGTGATGA-3 | 0.753 | 0.388 |
|  |  |  | r:5-TTTGACATGGGTATGCTTGTGA-3 |  |  |
|  |  |  | e:5-CTGACTGACTGACTGACTGACTGACTGAC |  |  |
|  |  |  | TAATGTTCGTTCTCGAGCGTGGTGA-3 |  |  |
| rs7319021 | A/C | intron | f:5-CTGTGACCTCATGGCATATACC-3 | 0.789 | 0.385 |
|  |  |  | r:5-ACCATTGCCTAATCCAAGATCG-3 |  |  |
|  |  |  | e:5-CTGACTGACTGACTGACTGACTCTTAGAA |  |  |
|  |  |  | GAAAACATAGGGATA-3 |  |  |
| **A2M** |  |  |  |  |  |
| rs10492115 | A/G | intron | f: 5-AAGAGCACCTAGACCCATG-3 | 0.053 | 0.138 |
|  |  |  | r:5-AAGAATCACTTGAGCCAGTAGA-3 |  |  |
|  |  |  | e:5-CTGACTGACTGACTGACTGACTGACTGAC |  |  |
|  |  |  | TCATGTCCCACCACAATTAAT-3 |  |  |
| rs669 | A/G | intron | f:5-TTGCTCCTAACATCTATGTACTGG-3 | 0.948 | 0.078 |
|  |  |  | r:5-CCTTACTCAAGTAATCACTCACCA-3 |  |  |
|  |  |  | e:5-CTGACTGACTGACTGACTGACTGACTGAA |  |  |
|  |  |  | ACACAGCAGCTTACTCCAGAG-3 |  |  |
| rs10842849 | A/G | intron | f:5-GTGGCATACACCTGTGGG-3 | 1 | 0.116 |
|  |  |  | r:5-CCACTTGACTAGGTAGATGATCC-3 |  |  |
|  |  |  | e:5-CTGACTGACTGACTGATAGCAGACTAAAT |  |  |
|  |  |  | CCAACAATACA-3 |  |  |
| rs11048839 | C/T | intron | f:5-GTGGCATACACCTGTGGG-3 |  |  |
|  |  |  | r:5-CCACTTGACTAGGTAGATGATCC-3 | 1 | 0.117 |
|  |  |  | e:5-CTGACTGACTGACACTTGAACTCCTGA |  |  |
|  |  |  | GTTCAAGCA-3 |  |  |
| rs10842847 | G/T | intron | f:5-ACATACTACCAAGACTGAATCATG-3 | 1 | 0.117 |
|  |  |  | r:5-CAGTGAAGCCAACAGTTATTAGG-3 |  |  |
|  |  |  | e:5-CTGACTGACTCTGATCTGAACTGAC |  |  |
|  |  |  | AAATAATGAGTAA-3 |  |  |
|  |  |  |  | (continued) | |
| **Table S1.** Descriptive Statistic of Genotyped Polymorphisms in the FoxO1, TGF-β1, and A2M Gene (continued) | | | | | |
| Markers | Alleles | Region | Primer Sets | HWE | MAF |
| rs226415 | A/G | intron | f:5-AAGTCTTTCAATTTTCCACTGTT-3 | 1 | 0.115 |
|  |  |  | r:5-CTCACCAAATTAGATATACAAGCA-3 |  |  |
|  |  |  | e:5-CTGACTGACTGACTGACTGACTGACTGAA |  |  |
|  |  |  | TACATGCCCTTTATTGTGTTGAG-3 |  |  |
| **TGF-β1** |  |  |  |  |  |
| rs12462166 | C/T | intron | f:5-CGTAGCTGGGGTGAGGAG-3 | 0.117 | 0.487 |
|  |  |  | r:5-CAGGGTGGCTGGGGAAAC-3 |  |  |
|  |  |  | e:5-CTGACGGGCTCCGCGAGCGATCCCCGCC-3 |  |  |
| rs12983775 | A/G | intron | f:5-CGTAGCTGGGGTGAGGAG-3 | 0.104 | 0.485 |
|  |  |  | r:5-CAGGGTGGCTGGGGAAAC-3 |  |  |
|  |  |  | e:5-CTGACTGACATGGGAAGGAAA |  |  |
|  |  |  | GGGAAGGGAGG-3 |  |  |
| rs1800469 | C/T | downstream | f:5-GGGAGGTGCTCAGTAAAGGA-3 | 0.185 | 0.485 |
|  |  | (500B) | r:5-GGGTGTCAGTGGGAGGAG-3 |  |  |
|  |  | upstream | e:5-CTGACTGACTGACTGACTGATCAGGTGT |  |  |
|  |  | (2KB) | CCTGTTGCCCCCTC-3 |  |  |
| rs2317130 | C/T | intron | f:5-AGCTGATATCCTAGACAACGAATG-3 | 0.129 | 0.487 |
|  |  | upstream | r:5-TTTCCCTCACAGCAATTACCA-3 |  |  |
|  |  | (2KB) | e:5-CTGACTGACTGACTGACTGACTGACTGAC |  |  |
|  |  |  | ACAGCAATTACCACCATCT-3 |  |  |
| rs2241715 |  | intron | f:5-CTGTCCACGCATGGGTCT-3 | 0.186 | 0.486 |
|  |  |  | r:5-GAGAGGCGGGGAGATGTC-3 |  |  |
|  |  |  | e:5-CTGACTGACTGACTGACTGACTTTCTATT |  |  |
|  |  |  | TTTCTCCTCCACGGTCC-3 |  |  |

Abbreviations: HWE, Hardy-Weinberg Equilibrium (p value of chi-square tests); MAF, minimal allele frequency; f, forward primers; r, reverse primers; e, extension primers.


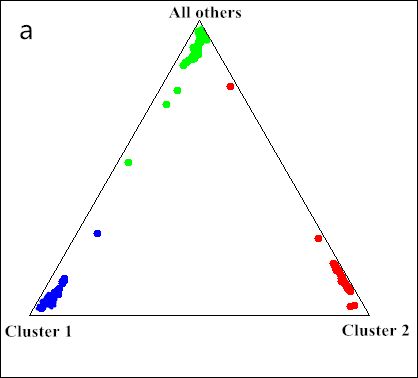

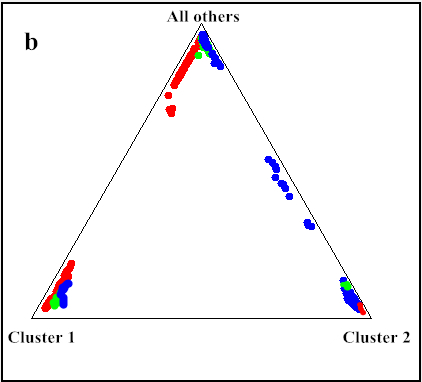


**Figure S1**.The triangle charts of population-stratification analysis when K=3.
